# Effectiveness and Safety of Intravenous Sedation with Propofol in Non-Operating Room Anesthesia (NORA) for Dental Treatment in Uncooperative Paediatric Patients

**DOI:** 10.3390/children8080648

**Published:** 2021-07-28

**Authors:** Gianmaria Fabrizio Ferrazzano, Tiziana Cantile, Martina Quaraniello, Michele Iannuzzi, Daniela Palumbo, Giuseppe Servillo, Silvia Caruso, Fabiana Fiasca, Aniello Ingenito

**Affiliations:** 1 Department of Neuroscience, Reproductive and Oral Science, School of Paediatric Dentistry, University of Naples ‘Federico II’, 80131 Naples, Italy; tizianacantile@yahoo.it (T.C.); martina.quaraniello@libero.it (M.Q.); ingenito@unina.it (A.I.); 2 Unesco Chair in Health Education and Sustainable Development, Paediatric Dentistry Section, University of Naples ‘Federico II’, 80131 Naples, Italy; 3 Department of Medicine, Surgery and Dentistry, Scuola Medica Salernitana, 84081 Salerno, Italy; 4 Department of Anesthesiology and Intensive Care Medicine, National Hospital of Sea, 80137 Naples, Italy; michele.iannuzzi74@gmail.com; 5 Department of Neuroscience, Reproductive and Oral Science, School of Anaesthesiology and Intensive Care Medicine, University of Naples ‘Federico II’, 80131 Naples, Italy; danielapalumbo00@gmail.com (D.P.); giuseppe.servillo@unina.it (G.S.); 6 Department of Health, Life and Environmental Science, University of L’Aquila, 67100 L’Aquila, Italy; silvia.caruso21@gmail.com (S.C.); fabiana.fiasca@alice.it (F.F.)

**Keywords:** uncooperative children, intravenous sedation, Propofol, NORA, paediatric dentistry, oral health

## Abstract

Background: Uncooperative children require sedative approach for dental treatment. The aim was to assess the effectiveness of Propofol in “Non-Operating Room Anesthesia” (NORA) for paediatric dental treatment; intraoperative side effects; postoperative side effects; post-discharge effects. Methods: a prospective study, involving 109 uncooperative children undergoing sedation in NORA using Propofol for dental treatment, was performed. Working sessions, success/failure, intraoperative and postoperative side effects, number of treatment; type of procedure were assessed. Parents completed a post-discharge questionnaire on: pain; crying; fever; vomiting; headache; drowsiness; excitability; irritability; ability to eat; drugs and medical care needing. Results: Success: 96.7%. Intraoperative side effects: 33.3%. Postoperative side effects: 6.4%. Statistically significant association between: intraoperative side effects and age (*p* = 0.001), health status (*p* = 0.0007), weight (*p* = 0.038), respectively; intraoperative side effects and number/ type of dental treatment (*p* = 0.0055) and scaling (*p* = 0.0001), respectively. For post-discharge questionnaires, statistically significant association between: age and crying (*p* = 0.0001) and headache (*p* = 0.002), respectively; health status and crying (*p* = 0.015) and drugs needing (*p* = 0.04), respectively; weight and crying (*p* = 0.0004); extraction and pain (*p* = 0.0001) and crying (*p*= 0.0073), respectively; scaling and crying (*p* = 0.04), excitability and irritability (*p* = 0.03), respectively. Conclusion: Propofol in NORA was effective with minimal side effects.

## 1. Introduction

Uncooperative children behavior makes dental treatment difficult and prejudices the quality of treatment provided; this causes a worsening of oral health with a higher incidence of untreated caries and more caries for uncooperative paediatric patients than their less anxious and more cooperative peers [1,2]. Furthermore, patients with particular medical conditions and with extensive dental complications tend to show greater fear and lack of cooperation, mainly due to physical problems, mental disabilities or behavioral management problems [3].

Therefore, uncooperative children need various patients’ management strategies to supply adequate treatment. The initial approach is to use behavioral management techniques, that are a set of procedures targeted at improving child’s cooperation, in order to obtain complete acceptance of dental care and, ultimately, decrease the child’s perception that the dental treatment is frightening [4]. 

Even though behavioral techniques could be useful to obtain children cooperation, some paediatric patients do not permit dental treatment and may require sedative approach [5,6].

Mild sedation (oral sedation with benzodiazepines and inhalation conscious sedation with nitrous oxide and oxygen) is the most commonly used procedure in uncooperative patients; however, the use of this sedation is not possible in all patients, especially in patients with severe psychomotor disabilities, because it requires a minimum of cooperation [7].

Intravenous (IV) sedation with Propofol in ‘Non-Operating Room Anesthesia’ (NORA) can be a valid alternative to achieve moderate sedation and to carry out dental treatment in totally uncooperative patients or in patients with special medical conditions [8]. Even though this anesthetic procedure is generally considered to be less popular in infants and children because of the challenge to gain vascular access, the availability of mixture of local anesthetics cream (to be applied before inserting the needle) and Propofol (hypnotic anesthetic) has renewed interest, especially for paediatric ambulatory surgery and for diagnostic procedures outside the operating room [9]. 

In fact, the increasing use of invasive diagnostic and therapeutic treatments, especially in uncooperative patients, has led to a larger request for anesthesia care in non-operating room location [10]. 

The ideal anesthetic technique for NORA dental treatment should allow fast onset and stable operating condition, while guaranteeing quick recovery of protective reflexes and of cognitive and psychomotor functions. Propofol is a well-known intravenous anesthetic for ambulatory procedures for its foreseeable recovery with minimum side effect profile [9].

Propofol is a γ-aminobutyric acid (GABA) receptor agonist and exerts its hypnotic action through potentiation of the effects of the inhibitory neurotransmitter GABA [11]. It binds to the β-subunit of the postsynaptic GABAA receptor, where it causes an inward directed chloride current that hyperpolarises the postsynaptic membrane and inhibits neuronal depolarisation. This effect is dose-dependent. At low concentrations, propofol potentiates GABA-activated inward chloride currents, while at higher concentrations, it directly activates the channel opening [11].

The pharmacokinetic profile of Propofol is incomparable and contributes to its advantageous characteristic. After an initial bolus, plasma levels rapidly decrease because of redistribution of Propofol from the brain into less perfused sites such as muscles and one of the rapid metabolic clearance occurs through the liver [11]. The recovery from its clinical effects is rapid, even after prolonged administration. In fact, the plasma Propofol concentration has to decline by only 10–20% to permit awakening [9].

However, some factors such as age, weight, preexisting medical conditions, medical therapy and type of surgical treatment could influence the Propofol dosage requirements. Since considerable inter-patient variability exists in both healthy and disable patients, it is necessary a careful titration of Propofol to minimise adverse effects, such as hypotension, while permitting a rapid recovery [9].

Recovery is rapid both after a single bolus and after a continuous infusion, which allows Propofol to be used to maintain anesthesia during short outpatient procedures [9].

Propofol has many pharmacological advantages when compared to other anesthetic agents, such as fast onset and offset time, and it can be titrated very accurately both by hand or by using target-controlled infusion (TCI) technology [12].

Thanks to its different characteristics compared to other sedatives, Propofol results in more effective sedation, better patient cooperation and quick awakening with a minimal frequency of side effects. Another advantage is fewer side effects due to its anti-emetic effect (less postoperative nausea and vomiting) and less emergence delirium. The mechanism behind the anti-emetic effect is not very well understood, but it has been demonstrated that propofol interacts with dopaminergic (D2) receptors in the chemoreceptor trigger zone, inhibits the limbic system, thereby interacting with cortical reflexes reaching the vomiting centre [13,14,15]. Disadvantages of propofol use include hypotension, apnea and airway obstruction [14].

When sedation with benzodiazepines is carried out, the specific antagonist, flumazenil, is always available for use in emergencies, such as accidental oversedation, iatrogenic overdose or paradoxical reactions. On the contrary, there is no antagonist for propofol, so it is essential to perform this sedative procedure in NORA with the presence of an anesthetist [15].

Furthermore, intravenous sedation with Propofol in NORA can be a useful alternative to general anesthesia (GA), especially in patient who must undergo short and non-invasive dental treatment.

In fact, orotracheal intubation, length of the dental treatment and discharge duration are the main differences between IV and GA procedures [14]. The described complications of orotracheal intubation comprise of: sore throat, laryngeal edema, hoarseness, nerve injury, aspiration of oral or gastric contents, superficial laryngeal ulcers and laryngeal granuloma [16].

When compared to patients treated under sedation, general anesthesia patients often have a slower recovery of cognitive function, more pain, longer time to home readiness and lower patient appreciation [17].

Even though some studies show the absence of complications in patients with special needs treated under GA [3], this is often associated with considerable cost and there is a disproportional increase in costs associated with dental treatment under general anesthesia respect to intravenous sedation in NORA [18,19].

Based on these observations, the primary goal of this study was to assess the effectiveness of intravenous sedation in NORA using Propofol for dental treatment in uncooperative paediatric patients (both healthy/fearful and disabled patients). The secondary outcome was related to patient safety, considering side effects.

## 2. Materials and Methods

This study was approved by the Research Ethics Committee of the “Federico II University” Faculty of Dentistry, Naples, Italy (PT n° 33/19) and was carried out by the Department of Neuroscience, Reproductive and Oral Sciences, School of Paediatric Dentistry, University of Naples “Federico II”, in collaboration with the Section of Anaesthesia and Intensive care, University of Naples “Federico II”, Naples, Italy.

The study lasted from March 2019 to December 2019.

This prospective study used a convenience sample of uncooperative children which includes all consecutive patients referred for intravenous sedation in NORA with Propofol for dental treatment at the Dental Clinic of the University Hospital of Naples “Federico II”, Naples, Italy.

An 80% power, with a standard error of five per cent, and confidence level of 95 per cent were considered; to cover non-response, the sample was increased of 10%; therefore, sample size was estimated at 109 children.

In accordance with the Declaration of Helsinki, a total of 109 uncooperative children (both healthy/fearful and disabled children) with American Society of Anesthesiologists status I or II, aged ≥ 4 years, undergoing dental treatment under moderate sedation with Propofol in NORA, were recruited. For these patients, the inhalation sedation with nitrous oxide and oxygen procedure and oral midazolam sedation were contraindicated, failed or cannot be performed.

The exclusion criteria were children aged < 4 years and children with American Society of Anesthesiologists status III, IV and V [20].

A few days before the treatment, parents were invited to accompany their children to the Dental Clinic of the University Hospital of Naples “Federico II”, Italy—Paediatric Dentistry Section—to perform blood test, electrocardiogram (ECG), paediatric, cardiological and anesthetic examinations.

As the procedures were carried out in NORA, the dental treatment was performed in day hospital, so the patient was treated and discharged during the same day.

The aim and the procedures of the study were exposed to parents and paediatric patients, and they were requested to fill a written consent to participate in the study.

Before the treatment, patients received premedication with 0.5 mg/kg of Midazolam orally, up to a maximum of 15 mg. In this way, any anxious reaction has been attenuated or eliminated.

Dental treatment was performed by a paediatric dentist and included scaling with ultrasonic instruments, conservative therapies and dental extractions. After Propofol infusion, for more invasive procedures, such as dental extractions, loco-regional anesthesia with mepivacaine with adrenaline 1:100,000 was also provided.

Intravenous sedation in NORA was conducted by skilled staff consisting of paediatric anesthesiologists and nursing who are aware of NORA, increasing security, efficacy and patient/parent appreciation.

The equipment of NORA includes the presence of a multi-parameter monitor, laryngoscope, sphygmomanometer, pulse oximeter, capnometer, defibrillator, anesthesia trolley, a reliable source of oxygen and an adequate source of suction. Furthermore, there is a surgical washing area, a filter room for access and a post-operative environment for controlling the parameters up to the patient’s discharge (recovery room).

Sedation maintenance was achieved by titrating the infusion of Propofol at the preferred level of sedation. In addition to the infusion, a bolus of 10–20 mg has been used when an increase of depth of sedation was indispensable.

Blood pressure, heart rate and arterial oxygen saturation were registered at baseline and during the dental procedure continuously. Hypotension episode was defined as systolic blood pressure of <100 mmHg, bradycardia as heart rate of <60 bpm and fluctuation of saturation as arterial oxygen saturation between 93–95%.

The patients should be drowsy but awaken to the slight nociceptive stimulus.

Intravenous sedation with Propofol lasted a maximum of 30 min in each working session.

The effectiveness of the sedation was registered at the end of the treatment on a dichotomous scale: YES = success: scheduled dental treatment was completed; NO = failure: scheduled dental treatment was not completed due to ineffectiveness of sedation or the onset of side effects.

The following data were collected: number of working sessions, success/failure, intraoperative and postoperative side effects, number of teeth treated and type of dental procedure executed. At the discharge, parents were asked to fill in at home a questionnaire on post-discharge effects during the next 12 h to evaluate: post-operative pain; crying; fever; vomiting; headache; drowsiness; excitability; irritability; ability to eat; need to administer drugs at home; need for medical care after sedation.

After dental treatment, paediatric patients were transferred to hospital rooms reserved for them with televisions and electronic games to ensure awakening and a less traumatic post-operative course and parents were advised to administer painkillers to their children in case of need, especially after dental extractions.

All data for each patient were stored in a computer, secured with a password and then analysed.

### Statistical Analysis

A descriptive statistic was used to analyse the characteristics of the study sample. The discrete and nominal variables were expressed in terms of frequencies and percentages. Sample was stratified for: success/failure, presence or not of intraoperative and postoperative side effects and post-discharge effects, respectively. Differences were evaluated and tested using the χ2 or Fisher test (as appropriate) for the qualitative variables, as the non-normal data distribution (Shapiro–Wilk test). All the tests used were bi-directional and the level of statistical significance was fixed at 5%. All statistical procedures were carried out using the Statistical Package for the Social Sciences (SPSS version 21.0 for Windows).

## 3. Results

109 patients (75 males and 34 females), aged between 4 and 17 years, underwent intravenous sedation with Propofol in NORA to perform dental treatment.

The average age is 8.36 ± 3.68. The study population was composed of 37 healthy patients and 72 disabled patients.

In 95 patients one working session was performed and in 14 patients 2 working sessions were performed (for a total of 123 working sessions). The overall success rate was 96.7% (119 out of 123; 4 working sessions failed).

Most patients needed 1.5–4.5 mg/kg/h of Propofol to achieve the desired level of sedation.

Intraoperative side effects occurred in 33.3% of cases and the most frequently reported was fluctuation of saturation (30.1%), followed by bradycardia (1.6%) and reflexogenic stimulus (1.6%).

There is no statistical association between intraoperative side effects and gender.

Statistical analysis shows that, in relation to intraoperative side effects, there is a statistically significant difference between patients aged 4–7 years and patients aged 8–17 (*p* = 0.001; power = 0.724); there is a statistically significant difference between healthy patients and disabled patients (*p* = 0.0007; power = 0.865); there is a statistically significant differences between patients with weight < 40kg and patients with weight ≥ 40 kg (*p* = 0.038; power = 0.917) (Table 1).

Postoperative side effects occurred in 7 of the total sessions (6.4%) with agitation on waking in 4 patients (3.6%) and headache in 3 patients (2.7%).

Statistical analysis shows that there is no statistically significant association between postoperative side effects and gender, age, health status, weight, respectively.

During the study, 428 procedures were performed: 175 conservative therapies, 214 dental extractions and 39 scaling.

The number of teeth treated on average, in each working session, is 3.28 ± 2.63.

There is no statistically significant association between both intraoperative and postoperative side effects and the number of teeth on which conservative therapy has been performed.

There is no statistically significant association between both intraoperative and postoperative side effects and the number of teeth extracted.

There is a statistically significant association between intraoperative side effects, in particular fluctuation of saturation, and scaling procedure (*p* = 0.0001; power=1.000), but not between postoperative side effects and scaling procedure (Table 2).

In 22.7% of cases (27 working sessions), the treatment was performed on the upper dental arch; in 12.6% of cases (15 working sessions) on the lower dental arch and in 64.7% (77 sessions) of the cases on both dental arches. In relation to both intraoperative and postoperative side effects, there are no statistically significant differences between treatment performed on the upper dental arch and treatment performed on the lower dental arch.

In relation to the survey filled in by the parents after dental treatment on post-discharge effects, 18.5% of the patients had pain, 21% cried, 10.9% had fever, 1.7% vomited, 13.4% had headaches, 46.2% showed drowsiness, 20.2% showed excitability, 21% showed irritability, 16.8% failed to eat normally, 18.5% needed to take drugs (painkillers). No patient had to undergo further medical treatment.

There are no statistically significant differences between males and females in relation to post-discharge effects.

There is a statistically significant association between post-discharge crying and age (*p* = 0.0001; power = 0.591) and between post-discharge headache and age (*p* = 0.002; power = 0.120).

There is an association between post-discharge crying and the patient’s health status (*p* = 0.015; power = 0.175) and between post-discharge need to take drugs and the patient’s health status (*p* = 0.04; power = 0.265).

There is a statistical association between post-discharge crying and weight (*p* = 0.0004; power = 0.235).

Table 3 summarises the statistical analysis of post-discharge effects and patient’s characteristics.

In relation to the type of dental treatment performed there is an association between: post-discharge pain and dental extraction (*p* = 0.0001; power = 0.796), between post-discharge crying and dental extraction (*p* = 0.0073; power = 0.513) and scaling (*p* = 0.0002; power = 0.359), respectively; post-discharge excitability and scaling (*p* = 0.0002; power = 0.183) and post-discharge irritability and scaling (*p* = 0.0002; power = 0.601).

Table 4 summarises the statistical analysis of the post-discharge effects in relation to dental procedures performed.

There is no association between post-discharge effects and dental arches treated.

## 4. Discussion

Paediatric dentistry has provided several pharmaceutical and non-pharmaceutical methods for controlling children’s behavior; however, non-pharmaceutical and conscious sedative methods may not work for extremely uncooperative children. Therefore, for patients with severe anxiety and for intellectually disabled patients, intravenous sedation is often required for dental procedures [21].

The possibility of carrying out dental treatment in NORA is a great advantage as it allows to carry out anesthetic procedures in a different environment from the operating room. This allows the patient, who has to undergo short and non-invasive dental treatment, to be treated in all phases by highly specialised personnel and to avoid a longer hospital stay.

Drugs utilised for intravenous sedation in paediatric patients, submitted to dental treatment in NORA, should display specific properties, comprising fast onset of action and capability to suppress patient movements during treatments. Furthermore, great recovery should be assured, comprising marginal respiratory and circulatory depression, short time to awakening and reduction of postoperative side effects.

Propofol, which activates inhibitory GABA receptors, ensures fast recovery and it should be considered appropriate for intravenous sedation in paediatric patients submitted to ambulatory dental treatment [21].

In addition, in paediatric patients, premedication drugs are frequently used to alleviate the fear of procedure, to make separation from parents easier and to perform a pleasant induction of intravenous sedation.

In the present study, Midazolam, a short-acting benzodiazepine that produces anxiolytic, amnestic, hypnotic, anticonvulsant and skeletal muscle relaxant effects, was used as premedication agent with good results [22].

In our study, there was a disproportion regarding the gender of the patients (75 males and 34 females). Some previous studies supported this data distribution, although it is not clear why the masculine gender often outnumbers the feminine gender [3].

The most reported intraoperative side effects in children undergoing intravenous sedation were respiratory problems (5.5–31.7%). We observed similar levels of fluctuation of saturation (30.1%) with Propofol, but never below than 93% [23].

In particular, during a longer dental treatment a higher dose of Propofol is often required. This can lead to deeper sedation which could more easily cause respiratory depression [24].

Another side effect related to the administration of Propofol alone or in combination with other drugs is bradycardia [23]. In our study, the incidence of bradycardia was 1.6%.

Moreover, intraoperative side effects occurred with a higher frequency in disabled patients. In fact, hypotension with initial Propofol bolus is a temporary and predictable event that is more frequent in patients with special medical condition [23]. As these patients experience stressful situations in a more accentuated way, it is necessary a close collaboration between dentists and anesthetists to draw up a tailored therapeutic plan [25].

In addition, intraoperative side effects occurred in 44.7% of cases in patients with weight > 40 kg and in 52.4% of cases in patients aged 8–17 years. These patients, in fact, require higher doses of Propofol to obtain the desired level of sedation with a greater risk of occurrence of side effects. Chidambaran et al. reported that administration of Propofol in severely obese adolescents caused an overdose associated with slow awakening, increased postoperative sleepiness and incidence of postoperative respiratory side effects [26].

This study also showed statistical association between scaling and intraoperative side effects, in particular fluctuation of saturation. During intravenous sedation with Propofol, in fact, the patient is not intubated and retains the protective airway reflexes, so the greater quantity of water nebulised by the scaler during this procedure can reach the patient’s airway and cause laryngospasm with subsequent fluctuation of saturation. It is, therefore, essential to implement a series of strategies to obtain a careful aspiration of the liquids dispensed by dental instruments through the use of high-speed aspirators used by paediatric dentists highly specialised in the management of the airways. However, a study compared the laryngeal response to rigid laryngoscopy manipulation in children under sevoflurane anesthesia versus Propofol. The incidence of laryngospasm in the first group was 26% versus 4% in the Propofol group [27].

Intravenous sedation with Propofol showed a very low percentage (6.4%) of the onset of postoperative side effects as shown also by studies in the literature [28].

Propofol, in fact, has an antiemetic effect which has reduced the presence of side effects such as vomiting and nausea. Moreover, Propofol showed a lower frequency of agitation upon awakening (4.8%) than sevoflurane used in general anesthesia (58%) [29]. Uezono et al. demonstrated that in preschool children submitted to a minor procedure, sevoflurane determines a greater incidence of emergence agitation respect to Propofol (38% vs. 0%) [30].

Chandler et al. demonstrated a lower incidence of emergency delirium (ED) after intravenous sedation with Propofol compared to general anesthesia with sevoflurane in children aged 2 to 6 years [31]. Furthermore, their results confirmed that intravenous sedation with Propofol exhibits better characteristics in terms of awakening, post-treatment recovery and emergency delirium.

In addition, GA is an expensive procedure performed by trained anesthetists in hospital, and it requires a preoperative time before intervention for both dental practitioners and caregivers [3].

The first 24 h after dental treatment can be characterised by the onset of side effects. In this regard, it is essential that parents are informed by the dentist about post-sedation side effects and their management [32].

There is an association between post-discharge effects (especially crying, headache, difficulty to eat and need to take drugs) with the age, health status and weight of the patients.

In fact, the youngest and disabled patients cried after treatment. This may be related to an amplified perception of the treatment carried out, in particular after dental extractions. In fact, in a study carried out by Ozer et al., greater agitation was noted after treatments concerning dental surgery than those concerning restorative procedures [33].

In this regard, rooms were suitable for the awakening of small patients who, following treatment, require a reassuring environment. In fact, in the present study, patients who underwent intravenous sedation with Propofol, after dental treatment, were transferred to hospital rooms reserved for paediatric patients with televisions and electronic games to ensure awakening and a less traumatic post-operative course.

There is also an association between dental procedure performed (in particular extraction) and onset of post-discharge effects after treatment (specifically: pain, crying and the need to take drugs). These post-discharge effects were related to the dental procedure, rather than to the characteristics of intravenous sedation. In fact, scientific studies confirm that Propofol provides better post-operative pain relief with a significant reduction in pain scores by 40% compared to other types of sedation [28].

Our findings should be interpreted in the context of study limitations: first of all, the impossibility of using the rubber dam. In fact, this can represent an obstacle in emergency maneuvers if side effects arise. In the event that fluctuation of saturation occurs, the patient needs oxygen administration and removal of the rubber dam can delay this maneuver. Therefore, in restorative treatment it is very important to use good saliva aspiration and highly qualified dental assistance in order to complete the treatment in the best possible way.

## 5. Conclusions

This study demonstrated that intravenous sedation with Propofol in NORA can be used successfully when the dental treatment is essential, and the child does not cooperate during treatment.

Intravenous sedation with propofol allows to safely and effectively carry out dental procedures in non-cooperative paediatric patients in an outpatient setting by personnel qualified in the management of any complications. This also provides a financially valuable option to general anesthesia for paediatric dental procedures.

However, there are few studies in the literature on the use of intravenous sedation in NORA with Propofol to carry out dental procedures; so this study could be considered a starting point to open new scenarios for further studies on the matter.

## Figures and Tables

**Table 1:** Statistical significance of intraoperative side effects in relation to the characteristics of the patients.

Variables	Number of Working Session	Intraoperative Side Effects	No Intraoperative Side Effects	*p*-Value
Age, *n* (%)				0.001 **
4–7 years old	64 (52%)	10 (15.6%)	54 (84.4%)	
8–17 years old	59 (48%)	31 (52.5%)	28 (47.5%)	
Health Status, *n* (%)				0.0007 *
Healthy	39 (31.7%)	5 (12.8%)	34 (87.2%)	
Disabled	84 (68.3%)	36 (42.9%)	48 (57.1%)	
Weight, *n* (%)				0.038 **
<40 kg	85 (69.1%)	22 (25.9%)	63 (74.1%)	
≥40 kg	38 (30.9%)	17 (44.7%)	21 (55.3%)	

*p*-value using χ^2^ ** or Fisher’s * test.

**Table 2:** Statistical significance of intraoperative side effects in relation to dental treatment.

Variables	*N*	Intraoperative Side Effects	No Intraoperative Side Effects	*p*-Value
Scaling, *n* (%)				0.0001 *
Yes	39 (31.7%)	36 (92.3%)	3 (7.7%)	
No	84 (68.3%)	4 (4.8%)	80 (95.2%)	

*p*-value using Fisher’s * test.

**Table 3:** Statistical analysis of post-discharge effects and patient’s characteristics.

Variables	Crying		*p*-Value	Headache		*p*-Value	Drugs		*p*-Value
	Yes	No		Yes	No		Yes	No	
Age, *n* (%)			0.0001 *			0.002 *			
4–7 years old	23 (35.9%)	41 (64.1%)		14 (21.9%)	50 (78.1%)				
8–17 years old	2 (3.4%)	57 (96.6%)		2 (3.4%)	57 (96.6%)				
Healthy status, *n* (%)			0.015 **						0.04 *
Healthy	13 (33.3%)	26 (66.7%)					1 (2.6%)	38 (97.4%)	
Disabled	12 (14.3%)	72 (85.7%)					12 (14.3%)	72 (85.7%)	
Weight, *n* (%)			0.0004 *						
<40 kg	24 (28.2%)	61 (71.8%)							
≥40 kg	1 (2.6%)	37 (97.4%)							

*p*-value using χ^2^ ** or Fisher’s * test.

**Table 4:** Statistical analysis of the post-discharge side effects in relation to dental procedures performed.

Variables	Extracted Teeth		*p*-Value	Scaling		*p*-Value
	Yes	No		Yes	No	
Pain, *n* (%)			0.0001 **			
Yes	29 (74.4%)	12 (14.3%)				
No	10 (25.6%)	72 (85.7%)				
Crying, *n* (%)			0.0073 **			0.0002 *
Yes	14 (35.9%)	11 (13.1%)		0	20 (23.8%)	
No	25 (64.1%)	73 (86.9%)		39 (100%)	64 (76.2%)	
Excitability, *n* (%)						0.0002 *
Yes				0	20 (23.8%)	
No				39 (100%)	64 (76.2%)	
Irritability, *n* (%)						0.0002 *
Yes				0	20 (23.8%)	
No				39 (100%)	64 (76.2%)	

*p*-value using χ^2^ ** or Fisher’s * test.

## Data Availability

The data presented in this study are available on request from the corresponding author for a reasonable reason.
